# A Systematic Review of Smartphone and Tablet Use by Older Adults With and Without Cognitive Impairment

**DOI:** 10.1093/geroni/igac002

**Published:** 2022-01-06

**Authors:** Samantha A Wilson, Paula Byrne, Sarah E Rodgers, Michelle Maden

**Affiliations:** 1 Institute of Population Health, University of Liverpool, Liverpool, UK; 2 Liverpool Reviews and Implementation Group, University of Liverpool, Liverpool, UK

**Keywords:** Assistive technology, Brain injury, Dementia, Memory aid

## Abstract

**Background and Objectives:**

A systematic review was conducted to explore the use of smartphones and tablet computers as cognitive and memory aids by older adults with and without cognitive impairment, specifically the effects of smartphone and tablet use on participants’ cognition and memory, and the barriers and facilitators to smartphone and tablet use for cognitive and memory support.

**Research Design and Methods:**

A systematic search of 6 key databases found 11,895 citations published between 2010 and 2021. Studies were included if they involved community-dwelling older adults with or without cognitive impairment arising from acquired brain injury, mild cognitive impairment, or dementia, and if they evaluated everyday smartphone or tablet device use for cognition, memory, or activities of daily living.

**Results:**

A total of 28 papers were included in the narrative synthesis. There was some evidence that the use of smartphones and tablets could aid cognitive function in older adults without cognitive impairment, particularly executive function and processing speed. There was modest evidence that smartphone and tablet use could support memory in both older adults without cognitive impairment and those with acquired brain injury and dementia.

**Discussion and Implications:**

Smartphones and tablets were seen by users as acceptable, enjoyable, and nonstigmatizing alternatives to conventional assistive technology devices; however, current use of smartphone and tablet devices is hindered by the digital literacy of older adults, a lack of accommodation for older adult users’ motor and sensory impairments, and a lack of input from clinicians and researchers. Much of the evidence presented in this review derives from case studies and small-scale trials of smartphone and tablet training interventions. Further research is needed into older adults’ use of smartphones and tablets for cognitive support before and after the onset of cognitive impairment in order to develop effective evidence-based smart technology cognition and memory aids.

**Translational Significance:** This systematic review explores how older adults with and without cognitive impairment employ everyday smartphone and tablet devices to support their cognition, memory, and activities of daily living. There was some evidence that smartphone and tablet use could aid cognitive and memory function, which was enhanced by preexisting familiarity with these devices and early adoption in older adults with cognitive impairment. Accessibility issues due to motor, sensory, and cognitive impairments can limit the adoption of smart devices in older adult populations. Professionals’ input and support using these devices during rehabilitation is key to integrating device use into users’ everyday life.

At present, there are over 55 million people worldwide living with dementia, and this number is expected to rise to 139 million by 2050 (World Health Organization, 2021). A key symptom of dementia is cognitive decline that is usually progressive and irreversible. There is currently no effective pharmacological treatment (Karakaya et al., 2013). Thus, there is increasing interest in nonpharmacological approaches that optimize physical health, cognition, activity, and well-being of people living with dementia.

Assistive technology (AT) has been recommended in clinical practice guidelines in the United Kingdom as an intervention to maintain and improve the quality of life of both patients and their caregivers (Daly Lynn et al., 2019). It can help patients with dementia to increase their safety, confidence, and independence, as well as to reduce behavioral and psychological symptoms and maintain cognitive and social functioning, but AT has very low adoption in practice (Cohen-Mansfield et al., 2005; Gitlin et al., 1996; Scherer, 2005; Thorpe et al., 2016). Problems include the novelty or complexity of AT for people with cognitive impairment (LoPresti et al., 2004) and mismatch between the user’s cognitive profile and the AT (De Joode et al., 2010). Mobile devices such as mobile phones, smartphones, and tablets are highly accessible forms of AT and are used more widely than personal computers and older portable electronic devices (De Joode et al., 2013; Hart et al., 2004; Jamieson et al., 2017; Menéndez Álvarez-Dardet et al., 2020; Wong, Sinclair et al., 2017). Smartphone and tablet devices appear to offer some benefits for enhancing the quality of life of people with dementia, especially enabling them to stay independent and socially engaged in the early phases of the disease (Thorpe et al., 2016).

Far from being limited to children and young adults, the smartphone revolution has also affected older adults. Smartphone ownership has risen from only 10% of older adults using smartphones in 2011 to 61% in 2021 (Pew Research Center, 2021). Smartphone and tablet technology is ideal for health care interventions because the devices include multiple features, such as Internet access, mobile telecommunications, sensors, geolocation data, notifications, the ability to install applications (apps) that are clinically focused (Putzer, 2012). Smartphones and tablets contain sensors that can provide support similar to dedicated assistive devices without the burden of carrying a separate device at all times or the stigma of more visible assistive devices (Shinohara & Wobbrock, 2011). Increasingly in the future, they will be familiar to users who already rely on these technologies in their everyday lives before the onset of cognitive impairment, making them easier to learn to use as AT (Cohen-Mansfield et al., 2005; Thorpe et al., 2016). Despite their permeation into modern society, rehabilitation practice has been slow to adopt these new technologies (De Joode et al., 2012).

Smart technology-based research for older adults began to rise in 2014, but there is still a lack of gerontological smart technology-based studies (Kim & Lee, 2017). This is a lost opportunity, as more older adults are interested in using smart technology devices (Menéndez Álvarez-Dardet et al., 2020; Pew Research Center, 2014). Case studies have shown that training people with dementia in the use of smartphones and tablet computers can be beneficial in maintaining activities of daily living (ADL; Bier et al., 2015, 2018; Imbeault et al., 2018). However, a 2017 review found that few studies have investigated the use of smartphones by people with dementia (Klimova, 2017). Furthermore, the studies included in this review discussed the use of specific apps and tracking systems developed by research teams as opposed to the day-to-day use of smartphones by people living with dementia. It is unclear whether these findings apply to people who are not participating in an intervention and have not been specifically shown how to use their smartphones to support their cognition and memory. Most studies so far have ignored the potential of self-initiated strategies for learning and problem-solving in people with mild cognitive impairment (MCI) and dementia (Rosenberg & Nygard, 2014, 2017). Understanding how people with cognitive impairment spontaneously use their smart devices is important as spontaneous use can be predictive of how effective an aid will be for cognitive rehabilitation (Sohlberg & Mateer, 2001). In the absence of a comparison group, it is also unclear how attitudes toward and experiences of smartphones in older adults with cognitive impairment may differ from those of cognitively healthy older adults.

This systematic review seeks to explore the patterns of use and effectiveness of smartphones and tablet devices to support cognition and memory in older adults with and without acquired brain injury (ABI), MCI, or dementia, and to explore the opportunities and challenges of these devices in these populations. It is thought that the use of mobile technologies to offer cognitive support could generalize to assist not only people living with dementia but also people experiencing similar cognitive limitations due to illnesses or disabilities such as stroke, mental illness, brain injury, and physical or sensory disability (Koo & Vizer, 2019). Although predominantly focused on dementia and MCI, due to the paucity of literature on this topic reported in the studies of Kim and Lee (2017) and Klimova (2017), the parameters of this review were expanded to include the use of smartphones and tablet devices by not only people with dementia and MCI but also older adults with cognitive impairment due to ABI. This review is interested in acquired cognitive impairment as opposed to the chronic cognitive impairment that may be experienced by people living with mental illness, physical or sensory disabilities. Older adults with ABI included in this review experienced a cognitive decline due to stroke or traumatic brain injury. Also included were cognitively healthy older adults as a comparison group. Evidence has shown that cognitively healthy older adults who have subjective cognitive complaints may be at the earliest identifiable stages of neurodegenerative processes, with 30% developing MCI within 7 years (Chen et al., 2017; Rabin et al., 2017). Thus, even individuals who do not demonstrate clinical impairment could benefit from understanding how to use their smartphone to build compensatory habits for normal age-related changes in cognition or early neurodegeneration (Benge et al., 2020; Borella et al., 2013; Klimova, 2016; Oh et al., 2018; Shin et al., 2017).

## Objectives

The current systematic review aims to systematically search published literature to answer the following questions:

1. How do older adults with and without cognitive impairment use smartphone and tablet devices to support cognition and memory?
2. What effect does the use of smartphone and tablet devices as cognitive and memory aids have on older adults with and without cognitive impairment?
3. What are the barriers and facilitators of smartphone and tablet use as cognitive supports in older adults with and without cognitive impairment?

## Method

The review followed the Preferred Reporting Items for Systematic Reviews and Meta-Analyses (PRISMA) guidelines for the reporting of systematic reviews (Page et al., 2020) and was registered with PROSPERO: registration CRD42020176865 (Shamseer et al., 2015).

### Inclusion Criteria

Studies were included if they involved community-dwelling older adults (aged ≥50 years) with or without cognitive impairment arising from ABI, MCI, or dementia. It is now accepted that the neurodegenerative process of Alzheimer’s disease begins in mid-life (Habib et al., 2017); therefore, a minimum age of 50 years was selected to broaden the scope of the search and include younger populations who may be more familiar with and more frequent users of smartphone and tablet devices.

Studies were included if they evaluated everyday smartphone or tablet device use for ADL, instrumental activities of daily living (iADL), cognition, or memory. For this review, “everyday smartphone and tablet devices” are defined as any low-cost, off-the-shelf, unmodified smartphone or tablet devices with native apps and/or commercially available apps installed (Evald, 2018; Szabo & Dittelman, 2014). Native apps are preloaded on mobile devices, such as calculator and calendar apps. Unlike commercially available apps, such as to-do list apps, they do not need to be downloaded separately. This definition of everyday smartphone and tablet device use does not include smartphone or tablet devices linked to smart home or wearable hardware, nor does this include smartphones or tablet devices with research-driven apps or software installed, for example, apps designed and developed by clinicians or researchers to support cognition, memory, ADL, or iADL. This software may not be readily accessible to the general population; therefore, studies evaluating such software were excluded.

Studies were included if they measured at least one of the primary outcomes of interest: patterns of smartphone and tablet device use to aid cognition, memory, ADL, or iADL; effects on cognition, memory, ADL, or iADL; and patient evaluations of smartphone and tablet devices as cognitive and memory aids. Additionally, studies were included if they explored barriers and facilitators to smartphone and tablet device use to aid cognition, memory, ADL, or iADL and any other benefits or harms of technology use. The secondary outcomes of interest were as follows: psychological functioning (e.g., anxiety, mood, self-esteem, and self-efficacy), social functioning or participation, and any other benefits or harms of technology use.

Due to the lack of efficacy trials available, this review was not limited to randomized controlled trials. Any primary studies using quantitative or qualitative methods (or both) were included, including case reports. As in the study of Koo and Vizer (2019), study protocols meeting the above criteria were included so that the review reflects the newest research trends.

### Exclusion Criteria

Studies conducted in clinical settings or residential care settings were excluded, as were studies involving participants younger than 50 years or participants with cognitive impairment arising from other medical conditions, for example, learning disabilities.

Studies that included everyday smartphone or tablet device use for assessment or diagnosis, health monitoring, physical activity monitoring, or tracking were excluded.

Due to resource availability, papers written in languages other than English were excluded. Papers published before 2010 were excluded to accommodate the introduction of tablets and the wide adoption of smartphones (Pew Research Center, 2014). Articles from before 2010 may use outdated technologies (e.g., personal digital assistants) and may be less relevant for a contemporary audience.

Studies solely evaluating the design and feasibility of the technology were excluded. Conference proceedings, editorials, letters, and reviews of any kind were excluded. Included studies were marked as full-text articles or protocols accordingly.

### Search Procedure

The following key electronic databases were searched: Cochrane Central Controlled Register of Trials, CINAHL Plus, MEDLINE OVID, PsycINFO, Scopus, and Web of Science. Search terms were adapted for each database. The strategy contained both MeSH terms and textwords to increase the reach of the search. Search terms were derived from keywords cited in relevant key papers, as well as from the MeSH Browser (Supplementary Table 1). The search took place in June 2021.

Database search results were downloaded and imported to reference management software EndNote X8. One reviewer screened all titles and abstracts and 10% were screened by a second reviewer to check the accuracy of the screening. Disagreement was addressed through discussion. Likewise, one reviewer screened all full papers, with a 10% check from a second reviewer. Forward and backward searching were conducted on included articles to check for other articles eligible for inclusion in this review. The Mixed Methods Appraisal Tool for systematic mixed-methods reviews was used to assess the quality of the studies selected for this review (Hong et al., 2018); however, articles were not excluded based on quality assessment. Articles were organized into “stoplight” categories of red, amber, and green to indicate the quality of the articles. Categorization was based on sample size and number of sites included in the study, where red was *n* = 1 in a single site, amber was *n* ≥1 in a single site, and green was multiple sites.

### Data Extraction and Synthesis

A custom data extraction form was created for this review to include items such as technology and outcome measures as well as information about the study such as authors, year of publication, sample demographics, data collection methods, data analysis, and qualitative data on participant evaluations of the devices. Due to the wide scope of this review and the heterogeneity of study designs, populations, technologies, and evaluation methods, not all results were compatible with each other. For example, it would have been useful to compare results based on participants’ Mini-Mental State Examination results; however, this measure was only collected in three of the included studies. Therefore, a narrative synthesis was conducted, and results were compared based on diagnosis between older adults without cognitive impairment and older adults with cognitive impairment arising from ABI, MCI, or dementia.

## Results

**Figure 1.**
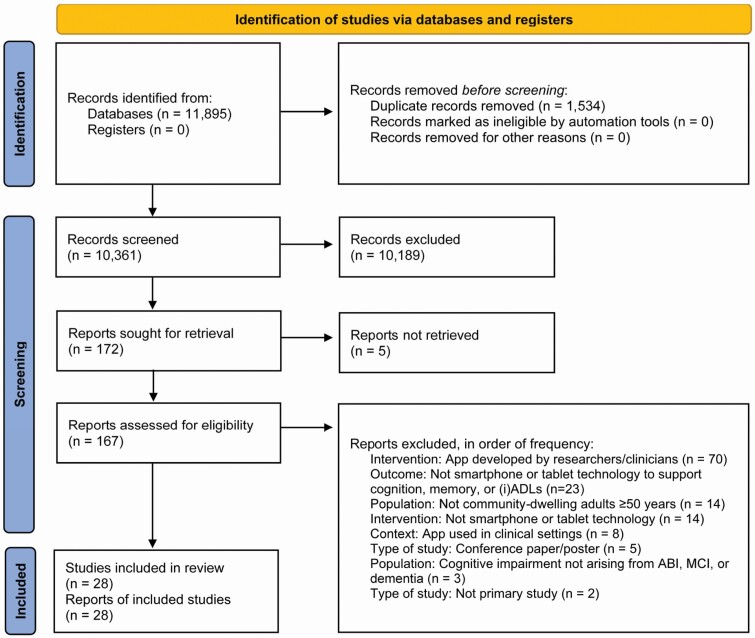
PRISMA flow diagram. (i)ADL = (instrumental) activities of daily living, *Note:* From Page et al. (2020).

The search results are displayed in Figure 1. A total of 11,895 records were identified. After removing duplicates and applying the inclusion and exclusion criteria, 172 articles were sought for retrieval and 167 full-text articles were obtained and reviewed. The references and citations of included papers were reviewed to check for eligible studies, which returned three additional papers. A total of 28 papers, including two study protocols, met the criteria and were included in the review (Table 1). Full results are given in Supplementary Table 2. A summary infographic produced in collaboration with people living with cognitive impairment is given in Supplementary Figure 1.

**Table 1.** Summary of Study Designs, Aims, and Results

Study; country	Study design, sample size, and clinical population (MMAT score)	Study aims	Key findings
*Acquired brain injury (n = 8)*			
Benge et al. (2020); United States	Cross-sectional survey. 53 adults with ABI (mean age 61 years), 44 care partners (mean age 54 years), control group of 40 older adults without cognitive impairment (mean age 54). (Green)	To investigate how patients referred for neuropsychological evaluations and their care partners use their smartphones and how these groups spontaneously use different features of their smartphones.	No statistically significant differences between patient, care partner, and control groups on use of social or general smartphone use. Cognitive aid features less commonly used across all groups. Patients and care partners reported using cognitive aid features significantly more often than the control group.
Bos et al. (2017); New Zealand^a^	Single-case series study. Five adults with ABI, mean age 52 years, range 25–63 years. (Amber)	To investigate the efficacy of a memory notebook and specifically a smartphone as a compensatory memory aid.	6 out of the 7 participants improved their ability to complete memory tasks when using the smartphone: more than when using the memory notebook. Smartphone audible reminders and greater portability were particularly beneficial. One participant showed a vast improvement in mood after the smartphone intervention that continued to improve at follow-up. Two other participants showed fluctuations in mood, but these were attributed to other factors.
Gustavsson et al. (2018); Sweden and Denmark^b^	Individual interviews 14 adults with ABI, mean age 61 years, range 41–79 years. Semistructured focus group interview. 4 adults with ABI, mean age 63 years, range 51–72 years. (Amber)	To identify how people 6–12 months after stroke were using and integrating ICT in their everyday lives.	Participants were motivated to use ICT: to feel safe, to be able to stay connected, to recreate and manage everyday life, and to solve obstacles to integrating ICT in daily activities. ICT can be used to practice cognitive and physical skills and participate in engaging activities that can aid recovery after stroke. Despite the impact of stroke on memory and thinking and fine motor skills, participants continued to use ICT 6–12 months after stroke.
Lemke et al. (2020); New Zealand	Observational and semistructured interviews. 6 adults with ABI (stroke), mean age 73 years, range 60–82 years. (Amber)	To describe the experience of ICT to explore the barriers and motivators to its use following stroke.	Participants used ICT devices to engage in daily activities and work tasks after stroke. Participants who felt comfortable and familiar with smart devices started to explore apps that could be downloaded to meet their needs. Two participants used tablets for rehabilitation on the recommendation of their physical therapist. None has been supported to explore problem-solving capabilities of ICT devices.
Ramirez-Hernandez et al. (2021); Australia^a^	Mixed-methods convergent design using quantitative and qualitative information collected in parallel during a similar time frame. 26 adults with ABI, mean age 62, range 40–79 years. (Amber)	To report the experience of participants and explore the acceptability of three training methods—trial-and-error, systematic instruction, and error-based learning—from the user perspective to inform the adoption of smartphone memory aid app training into clinical practice.	50% of interviewees reported that they were using their smartphones more in everyday activities, while approximately a third disagreed or strongly disagreed. Most participants found the smartphone training session enjoyable and became more familiar with their smartphones after the training. 46% considered one training session sufficient to learn to use the application, although others disagreed and strongly disagreed. 46% also reported that the training intervention encouraged them to explore new applications and devices.
Ramirez-Hernandez et al. (2020); Australia^b^	Three-armed phase II RCT. Older adults with ABI. (N/A)	To compare the efficacy of three training methods for training the use of a smartphone reminder app in ABI survivors presenting with memory complaints.	N/A
Rivest et al. (2018); Canada	Single-case experimental study using A-B-A-B time-series design. One adult with ABI (topographical disorientation), age 66 years. (Red)	To enable a man with topographical disorientation to navigate by foot or public transport without anxiety over getting lost.	A participant was able to efficiently and confidently use his smartphone apps to complete various wayfinding challenges. He self-initiated planning and preparations the evening before navigating unfamiliar outings. He reported less fear, frustration, and stress. Participant and his wife reported an increased quality of life related to greater autonomy.
Wong, Wang et al. (2017); Australia	Cross-sectional survey. 29 adults with ABI (stroke), mean age 60 years, and control group of 29 older adults without cognitive impairments, mean age 56 years. (Amber)	To investigate patterns of and perspective on smartphone use in people with stroke in comparison to healthy participants; to identify facilitators and barriers to smartphone use in people with stroke; and to examine associations between smartphone use and daily functioning, including self-reported sensory, motor, language, and cognitive functions, mood, and community integration.	Significantly fewer participants in the stroke group than the control group were smartphone users. Smartphone users in both groups had a similarly high frequency of use and similar broad patterns of the frequency of use of apps. Main benefits of smartphones were portability and convenience, connectivity with others, and access to the Internet. Memory aid and organization apps were the second most commonly used category of applications in both the stroke group and the control group. Frequent users of memory and organizational applications reported higher participation in work, study, and volunteer activities, although the difference was not statistically significant.
*Dementia or mild cognitive impairment (n = 10)*			
Bier et al. (2015); Canada	Single-case experimental study using A-B-A design. One adult with semantic dementia, age 55 years. (Red)	To determine whether procedural memory could be used to optimize learning of smartphone functions in a man with semantic dementia and to explore if smartphone use can help him relearn useful concepts.	Participant was able to learn to use the smartphone functions and retain this knowledge at a 6-month follow-up. He also learned six more functions by follow-up. No improvement on any semantic memory tasks.
Bier et al. (2018); Canada	Single-case experimental study using A-B-A design. One adult with semantic dementia, age 56 years. (Red)	To describe the compensation strategies that a man with semantic dementia spontaneously uses to manage everyday activities and his use of a smartphone as an external aid and to help him build knowledge on smartphone functions to expand on his compensation strategies.	Participant improved his existing app use and learned to effectively use smartphone functions. He retained this knowledge at a 6-month follow-up. He was particularly proud of his use of Evernote as a logbook to help him remember the names of objects to communicate with his wife and go grocery shopping.
El Haj et al. (2017); France	Single-case experimental study using A-B-A design. One adult with dementia (AD), age 66 years. (Red)	To investigate whether an external memory aid would alleviate prospective memory problems in a patient with AD.	Participant forgot 7/12 targeted events and 6/12 control events in the baseline phase. In the intervention phase, she forgot only 2/12 targeted events and 5/12 control events. She already used her smartphone to text her children and grandchildren. She occasionally used the map application on her smartphone when afraid of becoming lost. She preferred the discrete assistance of her smartphone over a paper calendar.
El Haj et al. (2021); France	Cross-sectional trial. 22 adults with dementia (AD), split between intervention group (*n* = 11, mean age 72 years), and paper-based calendar control group (*n* = 11, mean age 75 years). (Amber)	To investigate the effects of smartphone calendar applications on prospective memory in two groups of patients with mild AD.	Less omission of prospective events in the smartphone-based calendar group than in the paper-based calendar group, suggesting beneficial effects of using smartphone calendar applications on prospective memory.
Imbeault et al. (2018); Canada	Exploratory single-case study. One adult with dementia, age 65 years. (Red)	To test whether a person with AD can learn to use the calendar application on her tablet computer and assess the impact of using the tablet on memory-related tasks, mood, and caregiver burden.	Participants reported fewer problems with prospective memory and retrospective memory. Her ability to perform memory-related tasks increased following the intervention. She spontaneously started playing games on her tablet for cognitive stimulation and using contacts, notebooks, photo, and recipe apps, without assistance. She was proud of her tablet use, and it had a positive impact on her confidence. Caregiver burden remained absent or light throughout the study.
Köhler et al. (2021); Germany	Semistructured interviews. 14 adults with mild cognitive impairment or mild to moderate dementia (AD), mean age 71 years, range 58–86 years. (Amber)	To examine the mobility needs of people with mild to moderate dementia, and how these mobility needs can be supported by navigational assistance technology.	Nine participants would prefer their navigational assistive technology be on a smartphone, and one participant preferred a tablet computer. Participants listed a reminder function, emergency calls, calendar, timetable for public transport and location display, watch, navigation, GPS, notes, and alarm function as important system functions.
Kwan et al. (2020); China	Cross-sectional trial with observations and qualitative interviews. 16 adults with dementia, median age 79 years, control group of 30 older adults without cognitive impairment, median age 67 years. (Amber)	To explore the acceptability, feasibility, and usability of older people with mild dementia to use smartphone for wayfinding.	All participants were able to successfully initiate Siri by voice on their first attempt. Most participants were able to complete the wayfinding trial following the Maps application with no significant differences between groups. Participants with mild dementia needed significantly more time to complete the training and wayfinding trials. It was feasible and acceptable for people with dementia to use smartphones with voice navigation controls.
Routhier et al. (2012); Canada	Study 2: Single-case experimental study using A-B design. One adult with semantic dementia, age 51 years. (Red)	To measure the effectiveness of a smartphone intervention to compensate for word comprehension and naming difficulties.	A participant was able to use search engines on Internet apps or encyclopedia apps to find semantic information or pictures of words. He frequently used the Internet app on his smartphone to prepare activities for work and used Internet dictionaries more than the paper dictionary. He considered the smartphone fast, efficient, portable, accessible, and nonstigmatizing compared to the paper dictionary. His use of the smartphone over the weekend suggests that he enjoyed it and perceived its usefulness.
Scullin (2020); United States^b^	RCT. 52 adults with dementia or mild clinical impairment. (N/A)	To investigate whether smartphone technology or a memory strategy can be used to assist participants with prospective memory tasks, reduce memory burden, and improve independent functioning in participants with mild AD.	N/A
Wu et al. (2019); France	Cross-sectional survey. 323 adults with dementia (*n* = 84), MCI (*n* = 127) and older adults without cognitive impairment (*n* = 112), mean age 76 years, categorized into four groups according to the frequency of digital device use (daily use vs. nondaily use). (Amber)	To investigate cognitive function in relation to the use of a computer and a touchscreen device among older adults attending a memory clinic.	Most participants used at least one type of digital device daily. Over a third used a touchscreen device daily and most were also daily computer users. Participants who used both a touchscreen device and a computer daily performed better on executive function and mental flexibility than other groups. Participants with dementia who did not regularly use any digital device performed worse in several cognitive measures compared to participants using a digital device every day.
*No cognitive impairment (n = 10)*			
Chan et al. (2016); United States	RCT. 54 older adults without cognitive impairment (mean age 75 years), split between iPad intervention group (*n* = 18, mean age 75), social activities group (*n* = 18, mean age 75), and placebo group (*n* = 18, mean age 75). (Amber)	To test whether older adults who were computer novices can be trained to become proficient users of a tablet computer using the iPad, which can be flexibly employed to perform many tasks associated with daily living.	Improved performance on processing speed and memory in iPad intervention group compared with a social control and a placebo control. Although some individuals in the control groups also experienced some cognitive improvements, the iPad group showed significantly more improvement over time.
Gitlow (2014); United States	Nonexperimental survey. 82 older adults without cognitive impairment, mean age 79 years, range 60–97 years. (Amber)	To investigate what types of technology older adults are using, what they are doing with these technologies, what they would like to be doing with technology, and what barriers are preventing them from doing what they would like to do.	56% of older adults surveyed had cell phones; 17% had tablets; 35% of older adults did not need of want a cell phone; 49% for tablets. The top three reasons for cell phone use were personal calling (41%), voicemail (27%), and emergencies (26%). The top three reasons for tablet use were emailing (7%), web browsing (5%), and contact information (4%). For cell phones, 14% of participants wanted to learn to use appointment reminders and alarms, 9% wanted to use texting, and 7% wanted to use the calendar features. For tablets, 8% wanted to learn to use alarms and appointment reminders, 7% wanted to use shopping, and 4% wanted to use email and calendar features.
Nguyen et al. (2015); Australia	Cross-sectional survey. 153 older adults without cognitive impairment older than 65 years. (Amber)	To investigate how older people identify, select, and learn to use mobile communication technologies to enhance communication and safety and support independent living.	84% of respondents were technology users but only 3.3% reported using a smartphone. 44% of respondents were interested in trying out new products, devices, or services. Devices were used for emergencies or security purposes, to be reachable by family and friends, and for a sense of safety. Fewer respondents would use devices for information services, work, recreation, or education. 20% of respondents considered reminders as important to assist with daily life. 18% of respondents considered assistance with navigation as important, with male respondents and those married/partnered more likely to use this function.
Petrovčič et al. (2019); Slovenia	Population-based survey. 1,581 older adults without cognitive impairment, mean age 68 years, range 55–95 years. (Amber)	To explore factors predicting seniors’ interest in using three different types of assistive apps.	Mobile phones were used by 90% of respondents, and 84% used them daily. 27% of mobile phone users used a smartphone. More than 81% of respondents had heard about smartphones, with 61% among them having at least some familiarity with a smartphone. Respondents were reluctant to adopt Internet-based mobile services and apps. One in four individuals older than 55 years of age had never downloaded a smartphone application. Respondents reported a high interest in an SOS button, followed by ICE contacts, fall detection, and GPS navigation. There was less interest in video calling, physical activity monitors, and medication reminders.
Rosales and Fernández-Ardèvol (2016); Spain^c^	Quantitative study tracking mobile app usage: 238 smartphone users, mean age 39 years, range 20–76 years. Qualitative focus group study: 24 older adults without cognitive impairment, mean age 71 years, range 55–81 years. (Amber)	To analyze the use of smartphones by older adults.	Smartphones were a central part of participants’ everyday lives, even those who were initially reluctant to have a smartphone or were critical of others’ excessive smartphone use. Personal information management apps were used more frequently by older participants. Participants reported extensive use of note apps and calendar apps as memory aids. Some participants reported the use of reminder apps.
Vaportzis et al. (2018); Scotland	Postintervention questionnaire: 43 older adults without cognitive impairment, mean age 69 years, range 55–76 years. Postintervention semistructured focus group study: 14 older adults without cognitive impairment, mean age 68 years, range 65–75 years. (Amber)	To investigate older adults’ experience in the “Tablet for Healthy Ageing” intervention program to understand what they found helpful or unhelpful about the tablet training intervention.	Participants were confident that tablet training could have beneficial effects on mental abilities. Some participants reported feeling cognitively faster and having better memory or reasoning skills. Processing speed was significantly improved. Half the group thought that a tablet could have positive effects on other aspects of health and well-being, such as active and healthy aging. Most participants reported that it was likely or very likely that they would use a tablet in the future.
Vaportzis, Clausen et al. (2017); Scotland	Semistructured focus group study. 18 older adults without cognitive impairment, mean age 71 years, range 65–75 years. (Amber)	To investigate perceptions of, and barriers to, interacting with tablets in healthy older adults who were novice tablet users, and to explore the acceptability and usability of tablets as a potential tool to improve the health and well-being of older adults.	Participants wanted to use tablets to communicate better with younger generations. Some participants believed that learning to use a tablet could improve various skills and abilities, such as faster cognition and keeping their brains active. Others reported concerns that it would be harder to focus or that tablet use would deter them from using their memory because they would not need to remember events or facts. Most participants enjoyed the tablet experience and said they were likely to use a tablet in the future. Half requested to be included in the “Tablet for Healthy Ageing” intervention program.
Vaportzis, Martin et al. (2017); Scotland	Prospective RCT. 43 older adults without cognitive impairment, range 65–75 years, split between intervention group (*n* = 22, mean age 68 years) and no-contact control group (*n* = 21, mean age 70 years). (Amber)	To test the efficacy of a tablet computer training intervention to improve cognitive abilities of older adults and to investigate whether engaging with a new mentally challenging activity has cognitive benefits.	Improved performance on processing speed in the intervention group. No significant main effects or interactions for verbal comprehension, perceptual reasoning, or working memory. For participants in the stroke group, the smartphone’s use as a memory and organizational aid was the most significant benefit.
Yuan et al. (2019); China	Cross-sectional survey. 2,600 older adults without cognitive impairment, mean age 69 years, stratified by gender and categorized into three groups according to the number of smartphone functions used. (Amber)	To investigate gender differences in the use of smartphones and in cognitive ability, as well as the associations between smartphone use and general cognitive health and multidomain cognitive health.	Nearly 30% of the total participants were smartphone users, the majority of which were men. Both male and female frequent smartphone users were more likely to attain higher scores in all cognitive subdomains than infrequent and nonsmartphone users. Use of more smartphone functions was positively associated with general cognitive health and all subdomains except memory and orientation.
Zilberman et al. (2016); United States	Preintervention and postintervention semistructured interviews. 8 older adults without cognitive impairment, mean age 68. (Amber)	To evaluate changes in self-reported participation and satisfaction of performance of up to five (i)ADLs for older adults after an 8-week educational tablet training program.	The ADL most frequently addressed by the tablet training intervention was functional mobility. Next was communication management, and then health management and maintenance, which included developing and managing routines for health and well-being promotion such as physical fitness and medication routines. Other (i)ADLs: community mobility, financial management, shopping, and meal preparation and clean-up. Participants’ perceived performance on the (i)ADLs significantly improved following the intervention. Despite some initial difficulties, all the participants enjoyed using the tablet, used their tablet outside of the program, and would continue to use it in their daily lives.

*Notes:* MMAT = Mixed Methods Appraisal Tool; ABI = acquired brain injury; AD = Alzheimer’s disease; (i)ADLs = (instrumental) activities of daily living; GPS = global positioning system; ICE = in case of emergency; ICT = information and communications technology; MCI = mild cognitive impairment; N/A = not applicable; RCT = randomized controlled trial.

^a^Study was included because, although some participants were younger than 50 years, the mean age of the sample was older than 50 years.

^b^Protocol.

^c^Study was included because, although the mean age of the quantitative study sample was less than 50 years, all qualitative study participants were over 50 years.

Of the 28 papers, 25 were categorized as amber or red in the quality assessment due to their small sample sizes. Six papers were classified as red as these were case studies of single individuals with ABI or dementia. Though rich in detail, the results from these studies may not be reliably reproduced, nor can the conclusions from these papers be readily generalized to other similar populations with cognitive impairment. Nineteen papers were classified as amber. This category included one case series study of five individuals and two trials involving relatively small samples of 22 and 46 total participants. One paper was classified as green—a cross-sectional survey where responses were gathered from multiple memory clinics (Benge et al., 2020). Only seven of these studies included a control group or independent comparison group. The mostly medium quality of the included studies is indicative of the exploratory nature of much research on the application of smart devices for gerontology. Given this quality assessment and issues of replicability and generalizability, broad patterns can be observed on the effects of smartphone and tablet use on cognition, memory, and other psychological domains on these populations, but there is currently insufficient evidence to form robust conclusions.

The results are organized into sections based on the three questions explored by this systematic review: (a) Current and prospective smartphone and tablet use; (b) Effect of smartphone and tablet use on cognition, memory, and (i)ADLs; (c) Effect of smartphone and tablet use on psychological domains; and (d) Barriers and facilitators to smartphone and tablet use.

### Current and Prospective Smartphone and Tablet Use

Participants reported varied familiarity with smartphones and tablets across all studies. Where papers distinguished between younger and older participants, younger participants showed greater motivation to use smartphone and tablet devices—particularly for work-related activities by participants in employment (Nguyen et al., 2015)—and were more likely to integrate smartphone and tablet use into their daily lives. Petrovčič et al. (2019) suggested that seniors were reluctant to adopt smartphone or tablet devices; however, older adults were currently using smartphones and tablets in their everyday life. Older adults without cognitive impairment were interested in building upon their smartphone and tablet use to incorporate cognitive aid features (e.g., alarms, calendars, navigation aids, and reminders) in their everyday smartphone and tablet use.

Older adults with cognitive impairment show significantly higher use of cognitive aid features (Benge et al., 2020; Wong, Wang et al., 2017) and organization features (Wong, Wang et al., 2017) of smartphones and tablets than older adults without cognitive impairment. Fewer older adults with cognitive impairment than older adults without cognitive impairment were smartphone users; nevertheless, both groups had a similarly high frequency of smartphone and tablet use and similar broad patterns of app use (Wong, Wang et al., 2017).

Fifteen studies concerned smartphone and tablet training interventions. In all these studies, participants were able to learn to use smartphones and tablets, and participants’ overall experiences were extremely positive. Tablet devices in particular were perceived as easy to use by people of all ages regardless of their previous use of or familiarity with them (Gustavsson et al., 2018). Where the study populations were older adults with cognitive impairment, participants were able to retain this knowledge of smartphone and tablet functions (Kwan et al., 2020; Rivest et al., 2018; Routhier et al., 2012), with some participants even demonstrating long-term retention of this learning at follow-up 6 and 12 months after the training intervention, despite the impact of their impairment on their memory, cognition, and fine motor skills (Bier et al., 2015, 2018; Gustavsson et al., 2018; Imbeault et al., 2018).

Following the training interventions, participants continued to show interest in using the smartphone or tablet devices. In the study of Chan et al. (2016), all participants subsequently obtained a tablet device either as a gift or by purchasing one themselves, and in the works of Vaportzis, Clausen et al. (2017) and Vaportzis et al. (2018), most participants reported that it was either likely or very likely that they would use a tablet in the future. Of the participants in the work of Bos et al. (2017) six of the seven participants with ABI continued to use the smartphone after the intervention; the remaining participant had been reluctant to use the smartphone from the outset of the study.

Participants both with and without cognitive impairment were able to generalize the skills learned during the training interventions to other smartphone and tablet functions. They were able to find, install, and use other apps without instruction to meet their individual needs and personal preferences. In addition to trained smartphone functions, the participant in the study of Bier et al. (2018) favored the Evernote app and regularly used it for many purposes for which they had not been trained. The participant in the work of Imbeault et al. (2018) used the calendar app as a logbook and notebook and installed additional game and cognitive stimulation apps, contact apps, and recipe apps. The spontaneous use of other apps in the works of Bier et al. (2018) and Imbeault et al. (2018) led to feelings of pride and increased self-efficacy in these individuals with dementia.

### Effect of Smartphone and Tablet Use on Cognition, Memory, and (i)ADLs

Participants with cognitive impairment showed significantly higher use of cognitive aid features of smartphones and tablets than older adults without cognitive impairment (Benge et al., 2020; Wong, Wang et al., 2017). Wong, Wang et al. (2017) found that older adults with cognitive impairment who frequently used memory and organizational apps reported higher productivity than participants who did not frequently use these apps, although the difference was not statistically significant. Improvement in cognition was observed in eight studies. Qualitative data described that older adults without cognitive impairment reported feeling cognitively “faster” following a training intervention (Vaportzis, Clausen et al., 2017; Vaportzis et al., 2018). Pre- and postintervention assessments of different cognitive domains found that processing speed was the domain most frequently found to improve with smartphone and tablet use in older adults both with and without dementia (Chan et al., 2016; Vaportzis, Martin et al., 2017; Vaportzis et al., 2018; Wu et al., 2019; Yuan et al., 2019). Improvements in assessment of executive functions, mental flexibility, attention, and language were also observed in participants with and without dementia (Wu et al., 2019; Yuan et al., 2019). Yuan et al. (2019) found that older adults without cognitive impairment who used more smartphone functions reported greater improvements in all cognitive domains than those who used fewer smartphone functions. Despite significant effects in some cognitive domains, Vaportzis, Martin et al. (2017) reported no postintervention effects or interactions of tablet use on verbal comprehension or perceptual reasoning in cognitively healthy older adults.

Six studies found that memory abilities improved after participants with and without cognitive impairment integrated smartphone and tablet use into their everyday lives. In fact, participants with ABI in the work of Wong, Wang et al. (2017) reported that the smartphone’s use as a memory and organization aid was the most significant benefit of using a smartphone. In the case study of Imbeault et al. (2018) of a person living with dementia, the participant’s prospective and retrospective memory greatly improved as they documented their everyday life on their tablet. This improvement occurred despite their spouse’s reports of more general day-to-day memory problems at follow-up. Improvement in targeted prospective memory tasks in people living with dementia was also observed following smartphone interventions in the works of El Haj et al. (2017, 2021). Being able to cue prospective tasks in a smartphone device, which would then offer auditory, tactile, and visual notifications—as opposed to paper-based calendars that offer only visual cues when or if a person views the calendar—removed the cognitive load associated with prospective memory (El Haj et al., 2017, 2021). Conversely, studies by Vaportzis, Martin et al. (2017) and Yuan et al. (2019) found no significant effects or interactions on memory abilities in older adults without cognitive impairment.

Older adults with and without cognitive impairment reported using their smartphones and tablets to manage—or in the case of older adults with ABI, to recreate—their everyday life and activities. These activities included taking care of errands, paying bills, seeking information, staying connected with others, and staying home alone safely. Participants in employment used their smartphones and tablets to complete work tasks (Bos et al., 2017; Gustavsson et al., 2018). The ability to perform ADLs and iADLs promoted independence. One study of smartphone and tablet use by older adults with ABI demonstrated that participants sought apps to assist in their ADLs and therapy more than apps to augment their social lives (Gustavsson et al., 2018). Older adults without cognitive impairment were interested in apps to meet their safety needs and were less interested in apps to meet their ADLs and social needs (Petrovčič et al., 2019). In another study of tablet use by older adults without cognitive impairment, participants’ perceived performance on their (i)ADLs significantly improved following the tablet training intervention, as did their satisfaction with their performance of these (i)ADLs (Zilberman et al., 2016).

Where an (i)ADL was specifically targeted by the smartphone or tablet intervention, this activity was most commonly wayfinding, although this was only the case in two studies (Kwan et al., 2020; Rivest et al., 2018). Participants in the studies of Köhler et al. (2021) and Petrovčič et al. (2019) also desired smartphone and tablet technology to assist navigation. The use of a smartphone and tablet device to aid wayfinding was found to be feasible and acceptable in both studies (Kwan et al., 2020; Rivest et al., 2018), including in a trial with people with dementia in a major metropolitan area (Kwan et al., 2020).

### Effect of Smartphone and Tablet Use on Psychological Domains

Few studies reported on the effect of smartphone and tablet use on noncognitive psychological domains. However, these changes were consistently positive, including stable or improved mood in older adults with cognitive impairment (Bos et al., 2017; Imbeault et al., 2018), decreased caregiver burden (Imbeault et al., 2018), greater autonomy, less fear, frustration and stress, and improved quality of life (Rivest et al., 2018).

### Barriers and Facilitators to Smartphone and Tablet Use

The barriers and facilitators to smartphone and tablet use are given in Table 2, with a full description of the barriers and facilitators in each study given in Supplementary Table 3.

**Table 2.** Summary of Barriers and Facilitators to Smartphone and Tablet Use, in Order of Frequency

Barriers to smartphone and tablet use	Facilitators to smartphone and tablet use
1. Motor impairments (*n* = 8, 29%)	1. Perceived usefulness (*n* = 9, 32%)
2. Sensory impairments (*n* = 7, 25%)	2. Preexisting familiarity with computers, ICT, and smart devices (*n* = 8, 29%)
3. Device-specific complaints (*n* = 6, 21%)	3. Portability of device (*n* = 6, 21%)
4. Difficult to learn to use (*n* = 6, 21%)	4. Convenience of multiple functions in one, small device (*n* = 5, 18%)
5. Cognitive impairments (*n* = 4, 14%)	
6. Lack of knowledge and familiarity (*n* = 4, 14%)	5. Easy to use (*n* = 5, 18%)
7. Lack of instruction (*n* = 3, 11%)	6. Perception of smartphone as nonstigmatizing (*n* = 5, 18%)
8. Older age (*n* = 3, 11%)	7. Feeling of connectedness with others (*n* = 4, 14%)
9. Technology anxiety and technophobia (*n* = 3, 11%)	8. Enhanced independence and self-efficacy (*n* = 4, 14%)
10. Cost of device (*n* = 2, 7%)	9. Device use met individual needs (*n* = 4, 14%)
11. Dislike of smartphone (*n* = 2, 7%)	10. Engagement in training intervention (*n* = 3, 11%)
12. Lower level of education (*n* = 2, 7%)	11. Enjoyment of learning something new (*n* = 3, 11%)
13. Lack of confidence (*n* = 2, 7%)	12. Interest and willingness to learn to use (*n* = 3, 11%)
14. No perceived need/confidence in existing cognitive and memory abilities (*n* = 2,7%)	13. Motivation from family and friends (*n* = 3, 11%)
	14. Younger age (*n* = 3, 11%)
15. Overwhelming choice of devices (*n* = 2, 7%)	15. Access to information and Internet (*n* = 2, 7%)
16. Preexisting strategies to aid memory (*n* = 2, 7%)	16. Feeling of safety (*n* = 2, 7%)
17. Fear of addiction to technology (*n* = 1, 4%)	17. Higher level of education (*n* = 2, 7%)
18. Feelings of inadequacy in comparison to younger generations (*n* = 1, 4%)	18. Audible notifications (*n* = 1, 4%)
	19. Compatibility with lifestyle (*n* = 1, 4%)
19. Higher socioeconomic status (*n* = 1, 4%)	20. Being in employment (*n* = 1, 4%)
20. ICT functionality did not meet individual needs (*n* = 1, 4%)	21. Enjoyment of app use (*n* = 1, 4%)
21. Lack of interest (*n* = 1, 4%)	22. Low smartphone anxiety (*n* = 1, 4%)
22. Living alone (*n* = 1, 4%)	23. Older age (*n* = 1, 4%)
23. Perception of ICT as “cheating” (*n* = 1, 4%)	24. Physical features of device (*n* = 1, 4%)
24. Presence or suspicion of geriatric cognitive disorder (*n* = 1, 4%)	25. Positive attitude to technology (*n* = 1, 4%)
25. Speech impairments (*n* = 1, 4%)	26. Presence of chronic health conditions (*n* = 1, 4%)
	27. Sense of competence and mastery (*n* = 1, 4%)
	28. Versatility of device (*n* = 1, 4%)

*Note:* ICT = Information and communications technology.

The most frequently reported barriers to smartphone and tablet use by older adults both with and without cognitive impairment pertained to the accessibility of the devices. The effects of motor and sensory impairments and device-specific complaints (e.g., physical features of the device, busy interface, weak or unreliable signal) made smartphones and tablets difficult to learn to use and continue using in both older adults with and without cognitive impairment. The presence of cognitive impairments was the fifth most commonly cited barrier to smart technology use, reported in four studies. As above, older adults with cognitive impairment were less likely than those without cognitive impairment to use smartphones and tablets for cognitive and memory support. While cognitive, motor, and sensory impairments are not modifiable, adjustments can be made to smartphones and tablets to enable those with motor and sensory impairments to use these devices with greater ease. In the work of Wong, Wang et al. (2017), the primary concern for participants in the stroke group was the ease of use. Several participants in the stroke group with motor impairments reported that they preferred using iPads or tablets due to the large screen and better ease of use (Wong, Wang et al., 2017). Of the 28 papers included in this review, only one (Bos et al., 2017) utilized additional software (an app to increase the size of the keyboard) to make the smartphone easier to use by people with ABI. Six of the seven participants in this study were willing to continue using their smartphone as a memory aid following the intervention, and the participant who was not had been reluctant to use the smartphone from the outset. There are no mentions of motor or sensory problems as obstacles in this study, suggesting that, with small modifications to the smartphone’s user interface, it is feasible to accommodate for motor and sensory impairments.

Also commonly cited as barriers to smartphone use were a lack of confidence, familiarity and knowledge of smartphone and tablet devices, and technology anxiety. Participants often reported finding the devices difficult to learn to use at the outset of training interventions which could have discouraged them from continuing to use the device. However, participants’ engagement with the training interventions encouraged them to overcome this barrier. Following the training interventions, the vast majority of participants continued to show interest in using the smartphone or tablet devices. In the work of Chan et al. (2016), all 18 older adult participants subsequently obtained a tablet device either as a gift or by purchasing one themselves. Similarly, in the work of Vaportzis et al. (2018), most participants (including those in the control group) reported that it was either likely or very likely that they would use a tablet in the future. Participants in the study of Vaportzis, Clausen et al. (2017) reported the same. The overwhelmingly positive reception of training interventions and subsequent smartphone and tablet use at follow-up suggest that this barrier can be easily overcome with practice with the devices.

An unexpected barrier to smartphone and tablet use was higher socioeconomic status. One might assume that lower socioeconomic status would be the greater barrier to smartphone and tablet use due to the once-prohibitive costs of digital devices; however, the large variety of smartphone and tablet devices available at different price points means they are more accessible than ever. Petrovčič et al. (2019) found that older adults of a higher socioeconomic status were less eager to invest in smartphones and tablets as cognitive and memory aids because they had sufficient resources to cover the expense of conventional care. Similarly, those living alone show negative attitudes toward health-assistive apps on smartphones. This may be because they perceive smart devices as replacements or threats for the provision of in-person services. Conversely, they found that older adults of a lower socioeconomic status were more likely to use a smartphone or tablet to meet their health and social care needs, as a smartphone or tablet app may be a more affordable solution to conventional health and social care. They also found that older adults in employment were more likely to use a smartphone or tablet in this way due to the additional restriction of working hours on access to health and social care, particularly in situations that required immediate care assistance.

The most frequently reported facilitator to smartphone and tablet use by older adults both with and without cognitive impairment was a perceived need for cognitive and memory support and the usefulness of the devices to meet these needs. This drive to seek support through smartphone and tablet use helped to overcome barriers to information and communication technology use and aided in integrating smartphone and tablet use into participants’ daily lives. Smartphones and tablets were seen by older adults with cognitive impairment as convenient and nonstigmatizing solutions to many unmet needs. Smartphones and tablets are not novel assistive devices, and a plethora of software is readily available in addition to general smart device functions to assist in cognition, memory, and daily living: alarm apps, calendar apps, contact apps, entertainment and cognitive stimulation apps, navigation apps, note apps, reminder apps, and safety apps (e.g., to monitor falls). Participants with cognitive impairment who experienced enhanced independence and self-efficacy through the use of such apps described that this reinforced the desire to use their smartphone and tablet. Reluctance or refusal to use smartphone and tablet technology was a major predictor of smartphone and tablet nonuse (Bos et al., 2017; Lemke et al., 2020). Reluctance to engage with smart devices was also preceded by the perception of smartphones and tablets as products for younger generations more interested in technology and video games (Lemke et al., 2020) and more skilled at using the devices (Vaportzis, Clausen et al., 2017; Vaportzis, Martin et al., 2017).

Additionally, in older adults with cognitive impairment, preexisting familiarity with smartphone and tablet technology was an important facilitator to smartphone and tablet use after the onset of cognitive impairment. This preexisting familiarity and practice contributed to the benefit the participant was able to derive from the technology (El Haj et al., 2017). This familiarity was not considered a facilitator to smart technology use in cognitively healthy normal adults, further supporting the early adoption of smart devices following brain injury or after the onset of neurodegeneration. Conversely, the process of reconstructing one’s life after the onset of cognitive impairment, including learning how to do things in a new way, could lead to greater acceptance of technologies—such as smartphone and tablet devices—that a person may not have previously used (Gustavsson et al., 2018). The drive to use information and communication technologies after stroke was strong regardless of participants’ earlier experiences and the effects of the cognitive impairment on their memory, thinking, and fine motor skills.

## Discussion

Twenty-eight papers were included in this systematic review on the use and effects of smartphone and tablet devices as cognitive and memory aids on older adults with and without cognitive impairment. The results showed that although older adults were not currently using their devices to aid their cognitive and memory skills, they were interested in exploring this, especially older adults with cognitive impairment. Trials of smartphones and tablets to support cognition reported positive effects of smartphone and tablet use on cognition, particularly processing speed and executive function, in older adults both with and without cognitive impairment (Chan et al., 2016; Vaportzis, Martin et al., 2017; Vaportzis et al., 2018; Wu et al., 2019; Yuan et al., 2019).

Qualitative data described that the smartphone was a central part of everyday life of older participants, even those who were initially reluctant to have a smartphone or those who were critical of excessive smartphone use among younger people (Rosales & Fernández-Ardèvol, 2016). It is important to equip cognitively healthy older adults with the tools and training they need to build these habits for normal age-related changes in cognition or early neurodegeneration (Benge et al., 2020; Borella et al., 2013; Klimova, 2016; Oh et al., 2018; Shin et al., 2017), as it was shown that the benefits one can derive from smartphone and tablet use as a cognitive aid are influenced by familiarity and practice with smartphones and tablets (El Haj et al., 2017). Most participants in the included studies were not digital natives, evidenced by the training interventions that underpinned 15 studies. Therefore, smartphones and tablets were not part of their everyday lives before the trials or, in the case of older adults with cognitive impairment, before the onset of cognitive impairment. In the future, as adults for whom smart technology has been an integral part of their lives from a much earlier age and who have successfully integrated smartphones and tablets into their everyday lives for years if not decades, the effects of smartphone and tablet use to aid cognition and memory may be observed to be much greater.

The ubiquity of smartphone and tablet devices and the commonality of their use by people of all ages and functional status meant that smartphones and tablets were not seen as a special aid. Use of a smartphone or tablet did not draw attention to the user or the user’s impairments, thus these devices were seen as less stigmatizing than other ATs (El Haj et al., 2017; Gustavsson et al., 2018; Lemke et al., 2020; Routhier et al., 2012). Cost is considered to be a disadvantage when comparing smartphones and tablets to other low- or no-tech ATs. This barrier to smartphone and tablet use was reported in two studies (Nguyen et al., 2015; Vaportzis, Clausen et al., 2017). However, there was mixed evidence overall on the influence of cost on the uptake of smartphone and tablet devices as cognitive aids (Nguyen et al., 2015; Petrovčič et al., 2019; Vaportzis, Clausen et al., 2017). Rather than the price of the device being viewed as prohibitive, it appears that cost as a barrier to smartphone and tablet use is related to participants’ socioeconomic background, their access to conventional health and social care, and whether they owned a smartphone or tablet prior to their cognitive impairment or its use as a cognitive aid. If a person owned a smartphone or tablet prior to its use as a cognitive aid, cost would not be a barrier; however, its initial cost would still be a concern for people from lower socioeconomic backgrounds.

A disadvantage of smartphone and tablet devices being so readily available is the overwhelming choice of hardware (Imbeault et al., 2018; Lemke et al., 2020), which may alienate those who are unfamiliar with these devices. This, combined with anxiety about technology or technophobia, supports the need for input from professionals to assist older adults in selecting and using smartphone and tablet devices as cognitive aids. Many participants described the smartphone or tablet as hard to learn to use. Three participants in the study of Zilberman et al. (2016) reported that the tablet was hard to learn to use and reported difficulties switching between applications and remembering instructions from the tablet training sessions; however, these criticisms were presented retrospectively and followed with comments on finding or wanting to find a solution to the problem. Despite some initial difficulties, all the participants enjoyed using the tablet, utilized their tablet outside of the program in their daily lives, and would continue to use it. Although participants rarely explored commercial options, a range of software and hardware exists, such as voice commands and keyboards, which may support smart technology use by older people with and without cognitive impairment. Where software was installed on devices to meet participants’ sensory or motor impairment, participants did not report any difficulty using the devices (Bos et al., 2017). With further research and a greater understanding of how older adults with and without cognitive impairment spontaneously and effectively use these devices to support their cognition and memory, it will be possible to recommend products (including software and hardware) to meet the user’s specific accessibility needs.

Additionally, participants with ABI needed support when using a new device, particularly immediately after the onset of their cognitive impairment (Gustavsson et al., 2018). Support from family and friends was needed, both when introducing something new and when something unexpected happened. During rehabilitation following brain injury, participants struggled with a lack of instruction on the potential of their smartphone or tablet as a cognitive and memory aid (Lemke et al., 2020). In the work of Lemke et al. (2020), only two participants described using their devices for rehabilitation at the recommendation of their therapist, and no participant had been supported to explore the smartphone as an aid. Similarly, in the study of Wong, Wang et al. (2017), only 20% of participants with ABI reported that a rehabilitation therapist had suggested using a smartphone as an AT. Three participants had spontaneously used their smartphones to help them remember things for therapy, but only one participant had been assisted in doing so (Wong, Wang et al., 2017). This instruction or support may not necessarily be extensive, as 46% of participants in the work of Ramirez-Hernandez et al. (2021) considered a single training session sufficient to learn to use a memory aid app. About 50% of participants reported that they were using their smartphone more in general everyday activities following the training session, and 46% reported that the training session encourages them to explore new applications and devices, although approximately a third reported using their smartphone no more than before the training session (Ramirez-Hernandez et al., 2021). Smartphone and tablet devices are underutilized as rehabilitation tools, and further research is needed to explore how they can be optimized and implemented as assistive technologies or rehabilitation tools (Lemke et al., 2020; Ramirez-Hernandez et al., 2020, 2021).

Twenty-eight papers met the criteria of “everyday smartphone and tablet device use” and were included in this review. Papers were excluded if a smartphone or tablet device was linked to smart home or wearable hardware or if the paper explored research-driven apps or software. A total of 70 papers were excluded during the full-text screening because they evaluated hardware or software developed by researchers or clinicians. Despite this investment in therapeutic apps, most participants in Wong, Wang et al. (2017) did not use therapy apps because they had not heard of them or because they did not need them. Rosenberg and Nygård (2014) observed that most studies so far have ignored the potential of self-initiated strategies for learning and problem-solving in people with MCI and dementia. Similarly, Petrovčič et al. (2019) reported that there was a greater focus on caregiver needs through patient monitoring than on the needs of the persons living with cognitive impairment. This discrepancy between research interests and user needs must be resolved before helpful software and hardware can be effectively designed and implemented, starting with understanding how older people spontaneously use existing smartphone and tablet technology. The inclusion of two study protocols in this review (Ramirez-Hernandez et al., 2020; Scullin, 2020) shows that researchers are now starting to explore how older adults use existing smart technologies to support cognition and memory in case of ABI and dementia.

### Limitations

Although their pathways to care are different, the lack of previous research on smartphone and tablet use in people with dementia necessitated that this review includes studies with older adults with cognitive impairment arising from different etiologies. This review included 10 papers on smartphone and tablet use by people with dementia or MCI (of which five studies were case studies) and eight papers on smartphone and tablet use by older adults with ABI (of which, two studies were case studies). Broad conclusions can be drawn on the effects of smartphone and tablet use on cognition, memory, and other psychological domains on these populations, but there is currently insufficient evidence to form meaningful conclusions for each population.

In 15 studies, participants were involved in smartphone and tablet training interventions because participants were not digital natives. As discussed above, this limits the potential beneficial effects they could derive from smartphone and tablet use (El Haj et al., 2017). In the future, as technology develops and the population becomes generally more digitally literate and familiar with new technologies, older adults will experience very different barriers and facilitators to smartphone and tablet use. Thus, the relevance of the included studies and the generalizability of this review to contemporary audiences will be limited. For example, it is likely that fewer people will find the devices unfamiliar and difficult to learn to use; and instead, their preexisting practice with and positive attitude toward smartphone and tablet technology will serve as powerful facilitators to future use. However, the smart device market is seeing exponential growth; therefore, the overwhelming choice of devices may present a greater barrier to uptake than reported here.

## Conclusion

This systematic review aimed to explore three research questions:

1. How do older adults with and without cognitive impairment use smartphone and tablet devices to support cognition and memory?

Smartphones are part of the everyday lives of today’s older adults and will become more important as the general population ages and the “older adult” demographic grows to include people for whom smartphones and tablets have been an integral part of their lives for decades. Despite their ubiquity, these devices are currently underutilized to support cognitive and memory problems, both by users and clinicians.

2. What effect does the use of smartphone and tablet devices as cognitive and memory aids have on older adults with and without cognitive impairment?

There is evidence that the use of smartphones and tablets can aid cognitive function, particularly executive function and processing speed, and modest evidence that smartphone and tablet use can support memory. Smartphones and tablets are seen by users as acceptable, enjoyable, and nonstigmatizing alternatives to conventional AT devices; however, current use of smartphone and tablet devices is hindered by the digital literacy of older people and an associated lack of input from clinicians and researchers.

3. What are the barriers and facilitators of smartphone and tablet use as cognitive supports in older adults with and without cognitive impairment?

Major barriers included cognitive, motor, and sensory impairments and device-specific complaints making these devices difficult to use without adjustments, but this barrier could be overcome with careful consideration and support from clinicians. Another common barrier of anxiety around technology or technophobia could be overcome through training interventions and continued practice with the devices. In practice, smartphone and tablet training interventions resulted in participants continuing to use their donated devices or purchasing their own postintervention. Facilitators included the perceived need and usefulness of smartphone and tablet devices to support cognition and memory and a drive to seek cognitive support through smartphone and tablet use. Smartphones and tablets were seen as convenient and nonstigmatizing solutions to many unmet needs. The barriers and facilitators to smartphone and tablet use discussed in this review are derived from a relatively small population of nondigital natives and, as both the populations and technologies age and develop, these factors may change greatly; therefore, conclusions cannot necessarily be extrapolated to these future populations.

There is a lack of gerontological smart device-based research (Kim & Lee, 2017) despite a growing interest in smartphone and tablet use for health management from older adults (Pew Research Center, 2014). Although there is interest in developing hardware and software targeted at people with cognitive impairment, most studies so far have ignored or underemphasized working with older adults to discuss and build on their lived experiences with smartphones and tablets. Though there is a lack of efficacy trials in the included literature, researchers are now exploring how older adults use existing smart technologies to remediate cognitive and memory problems (El Haj et al., 2021; Kwan et al. 2020; Ramirez-Hernandez et al., 2020, 2021; Scullin, 2020). Further research is needed into older adults’ spontaneous smartphone and tablet use before we can develop effective smart technology-based cognition and memory aids for older adults.

## Supplementary Material

igac002_suppl_Supplementary_Material
