# Supplementary material for: A Systematic Review of Smartphone and Tablet Use by Older Adults With and Without Cognitive Impairment
Source: Innov Aging. 2022 Jan 6;6(2):igac002. doi: 10.1093/geroni/igac002 (PMC8889997; doi:10.1093/geroni/igac002)
Supplement: igac002_suppl_Supplementary_Material [file igac002_suppl_supplementary_material.docx]

**Online Supplementary Material**

Supplementary Table 1: MEDLINE search criteria

| **Search Section** | **Search Terms** | **Boolean Operator** | |
| --- | --- | --- | --- |
| **Part 1:**  **Dementia/MCI** | exp dementia; exp Alzheimer’s disease; Alzheimer*; “mild cognitive impair*”; MCI; exp cognitive dysfunction; “cognitive impair*” | OR | AND |
| **Part 2:**  **ABI** | “acquired brain injur*”; ABI; “traumatic brain injur*”; TBI; brain injur*; head injur*; craniocerebral trauma; brain damage; exp stroke | OR |  |
| **Part 3:**  **Older adults** | exp aged; exp “aged, 80 and over”; ageing; aging; older adj adult*; older adj person*; older adj people*; pensioner*; elder* | OR |  |
| **Part 4:**  **Smartphone/tablet technology** | exp smartphone; smartphone*; “smart phone”; exp computers, handheld; “tablet computer”; “tablet device”; ipad*; “i pad*”; “i phone*”; iphone*; “smart technolog*” | OR |  |

Supplementary Table 2: Full summary of study characteristics and results

| **Study; country** | **Study design, sample size and clinical population (MMAT score)** | **Study aims** | **Technology** | **Outcome measures** | **Key findings** |
| --- | --- | --- | --- | --- | --- |
| **Acquired brain injury (n=8)** | | | | | |
| Benge et al. (2020) United States | Cross-sectional survey.  53 adults with ABI (mean age 61 years), 44 care partners (mean age 54 years), and normative control group of 204 adults without cognitive impairment (mean age 35 years) where the oldest 40 participants were used as an age-matched control group (mean age 54).  (Green). | To investigate how patients referred for neuropsychological evaluations and their care partners use their smartphone and how these groups spontaneously use different features of their smartphones. | Any smartphone use. | Self-reported smartphone utilisation data. | - No statistically significant differences between patient, care partner and control groups on use of social or general smartphone use. - Cognitive aid features less commonly used across all groups. - Patients and care partners reported using cognitive aid features significantly more often than the control group. |
| Bos et al. (2017) New Zealand ^b^ | Single-case series study.  Five adults with ABI, mean age 52 years, range 25 to 63 years.  (Amber). | To investigate the efficacy of a memory notebook and specifically a smartphone as a compensatory memory aid. | Android LG Optimus One p500. Address book applications (GoContacts EX), calendar applications (CalendarSnooze, Google Calendar, SimpleCalendar), reminder applications (CalendarSnooze), to-do list applications (GTasks, SimpleCalendar Widget), and SlideIT application to increased keyboard size. Smartphone training intervention for 2 weeks. | Functional memory tasks to be completed every week throughout baseline, intervention period and post-intervention, and task competition log to be completed every day throughout baseline, intervention period and post-intervention to monitor memory performance.  Interview data collected throughout data collection period. | - 6 out of the 7 participants improved their ability to complete memory tasks when using the smartphone: more than when using the memory notebook. Smartphone audible reminders and greater portability were particularly beneficial. - One participant showed a vast improvement in mood after the smartphone intervention which continued to improve at follow-up. Two other participants showed fluctuations in mood but these were attributed to other factors. |
| Gustavsson et al. (2018) Sweden and Denmark ^b^ | Individual interviews.  14 adults with ABI, mean age 61 years, range 41 to 79 years.  Semi-structured focus group interview.  4 adults with ABI, mean age 63 years, range 51 to 72 years.  (Amber). | To identify how people 6-12 months after stroke were using and integrating information and communication technology in their everyday lives. | Any smartphone and tablet use. | Demographic data gathered through health care records.  Domain scores (strength, memory and thinking, emotion, communication, ADL/iADL, mobility, hand function, and social participation) calculated using algorithm to gather information on the perceived impact of stroke in everyday life.  Open-ended interview questions and focus group with photos of different devices to gather rich qualitative data. | - Participants were motivated to use ICT: to feel safe; to be able to stay connected; to recreate and manage everyday life, and to solve obstacles to integrating ICT in daily activities. - ICT can be used to practice cognitive and physical skills and participate in engaging activities which can aid recovery after stroke. - Despite the impact of stroke on memory and thinking and fine motor skills, participants continued to use ICT 6–12 months after stroke. |
| Lemke et al. (2019) New Zealand | Observational and semi-structured interviews.  6 adults with ABI (stroke), mean age 73 years, range 60 to 82 years (Amber). | To describe the experience of information and communication technology to explore the barriers and motivators to its use following stroke. | Any smartphone and tablet use. | Semi-structured interviews to explore the type of ICT used by participants, the tasks undertaken using ICT in both everyday activities and rehabilitation, and barriers to ICT use. | - Participants used ICT devices to engage in daily activities and work tasks after stroke. - Participants who felt comfortable and familiar with smart devices started to explore apps that could be downloaded to meet their needs. - Two participants used tablets for rehabilitation on the recommendation of their physical therapist. None has been supported to explore problem-solving capabilities of ICT devices. |
| Ramirez-Hernandez et al. (2021) Australia ^b^ | Mixed methods convergent design using quantitative and qualitative information collected in parallel during a similar timeframe.  26 adults with ABI, mean age 62, range 40-79 years.  (Amber). | To report the experience of participants and explore the acceptability of three training methods – trial-and-error, systematic instruction and error-based learning – from the user perspective to inform the adoption of smartphone memory aid app training into clinical practice. | Smartphone. Reminder applications (Cozi Family Organiser, 24meLtd., Wunderlist). Smartphone training intervention for 1 week using either systematic instruction, error-based learning and trial-and-error training methods. | Rating questionnaire on enjoyment, duration and difficulty of training session, and effects on smartphone use and familiarity.  Semi-structured interview to prompt more in-depth information regarding their experience. | - 50% of interviewees reported that they were using their smartphone more in everyday activities, while approximately a third disagreed or strongly disagreed. - Most participants found the smartphone training session enjoyable and became more familiar with their smartphone after the training. 46% considered one training session sufficient to learn to use the application, although other disagreed and strongly disagreed. 46% also reported that the training intervention encouraged them to explore new applications and devices. |
| Ramirez-Hernandez et al. (2020) Australia ^a^ | Three-armed phase II RCT.  Older adults with ABI.  (N/A). | To compare the efficacy of three training methods for training the use of a smartphone reminder app in ABI survivors presenting with memory complaints. | Smartphone. Reminder applications (Cozi Family Organiser, 24meLtd., Wunderlist). Smartphone training intervention for 1 week using either systematic instruction, error-based learning and trial-and-error training methods. | Data on cognitive function, functioning in ADLs, and smartphone ability.  Proficiency of performance determined by the total number of correct tasks independently completed by the participant in the Cozi app after the training intervention at three time-points: immediately after the training, and 1- and 6- weeks after.  Data on frequency of smartphone use, confidence in smartphone use, generally smartphone usage and memory self-efficacy at endpoint. | N/A |
| Rivest et al. (2018) Canada | Single-case experimental study using ABAB time-series design.  One adult with ABI (topographical disorientation), age 66 years.  (Red). | To enable a man with topographical disorientation to navigate by foot or public transport without anxiety over getting lost. | iPhone. Compass application (Compass), map application (Apple Maps). | Performance on real-life navigational tasks and confidence ratings in ability to navigate various scenarios at each study stage.  Naturalistic observations during real-life navigational tasks to evaluate actual ability to navigate among different unknown city locations. | - Participant was able to efficiently and confidently use his smartphone apps to complete with various wayfinding challenges. - He self-initiated planning and preparations the evening before navigating unfamiliar outings. - He reported less fear, frustration, and stress. - Participant and his wife reported an increased quality of life related to greater autonomy. |
| Wong et al. (2017) Australia | Cross-sectional survey.  29 adults with ABI (stroke), mean age 60 years, and control group of 29 older adults without cognitive impairments, mean age 56 years.  (Amber). | To investigate patterns of and perspective on smartphone use in people with stroke in comparison to healthy participants; to identify facilitators and barriers to smartphone use in people with stroke, and to examine associations between smartphone use and daily functioning, including self-reported sensory, motor, language, and cognitive functions, mood, and community integration. | Any smartphone use. | Survey data on smartphone use and application use.  Data on daily functioning, sensory function, motor function, language, cognitive function, mood, and community integration. | - Significantly fewer participants in the stroke group than the control group were smartphone users. Smartphone users in both groups had a similarly high frequency of use, and similar broad patterns of the frequency of use of apps. - Main benefits of smartphones were portability and convenience, connectivity with others, and access to the Internet. - Memory aid and organisation apps were the second most commonly use category of applications in both the stroke group and the control group. - Frequent users of memory and organisational applications reported higher participation in work, study and volunteer activities, although the difference was not statistically significant. |
| **Dementia or mild cognitive impairment (n=10)** | | | | | |
| Bier et al. (2015) Canada | Single-case experimental study using ABA design.  One adult with semantic dementia, age 55 years.  (Red). | To determine whether procedural memory could be used to optimise learning of smartphone functions in a man with semantic dementia, and to explore if smartphone use can help him relearn useful concepts. | Smartphone, Internet application. | Smartphone function task at baseline and post-intervention to assess ability to complete steps related to smartphone functions. | - Participant was able to learn to use the smartphone functions and retain this knowledge at 6-month follow-up. - He also learned 6 more functions by follow-up. - No improvement on any semantic memory tasks. |
| Bier et al. (2018) Canada | Single-case experimental study using ABA design.  One adult with semantic dementia, age 56 years.  (Red). | To describe the compensation strategies that a man with semantic dementia spontaneously uses to manage everyday activities and his use of a smartphone as an external aid, and to help him build knowledge on smartphone functions to expand on his compensation strategies. | Smartphone. | Smartphone function task at baseline and post-intervention to assess ability to use smartphone functions. Interview and observation data to record day-to-day compensation strategies. | - Participant improved his existing app use and learned to effectively use smartphone functions. - He retained this knowledge at 6-month follow-up. - He was particularly proud of his use of Evernote as a logbook to help him remember the names of objects to communicate with his wife and go grocery shopping. |
| El Haj et al. (2017) France | Single-case experimental study using ABA design.  One adult with dementia (AD), age 66 years (Red). | To investigate whether an external memory aid would alleviate prospective memory problems in a patient with AD. | Smartphone. Calendar application (Google calendar). | Performance on three targeted prospective memory events cued by Google calendar versus control (non-cued) events.  Data on cognitive ability, verbal episodic memory, working memory, mood, prospective memory, and Likert scale to measure participant’s experience with the Google calendar interview. | - Participant forgot 7/12 targeted events and 6/12 control events in the baseline phase. In the intervention phase, she forgot only 2/12 targeted events and 5/12 control events. - She already used her smartphone to text her children and grandchildren. She occasionally used the map application on her smartphone when afraid of becoming lost. - She preferred the discrete assistance of her smartphone over a paper calendar. |
| El Haj et al. (2021) France | Cross-sectional trial.  22 adults with dementia (AD), split between intervention group (n=11, mean age 72 years) and paper-based calendar control group (n=11, mean age 75 years).  (Amber). | To investigate the effects of smartphone calendar applications on prospective memory in two groups of patients with mild AD. | Smartphone. Calendar applications (Google calendar and Outlook calendar). Smartphone-based and paper-based tool training intervention for 2 weeks. | Performance on three targeted prospective memory events.  Data on cognitive functioning, working memory, and episodic memory. | - Less omission of prospective events in the smartphone-based calendar group than in the paper-based calendar group, suggesting beneficial effects of using smartphone calendar applications on prospective memory. |
| Imbeault et al. (2018) Canada | Exploratory single-case study.  One adult with dementia, age 65 years.  (Red). | To test whether a person with AD can learn to use the calendar application on her tablet computer, and assess the impact of using the tablet on memory-related tasks, mood and caregiver burden | Tablet. | Performance on three targeted prospective memory tasks versus seven control tasks at baseline and post-intervention.  Data on cognition, memory, mood and caregiver burden at baseline and post-intervention.  Observation journals throughout data collection period. | - Participant reported fewer problems with prospective memory and retrospective memory. Her ability to perform memory-related tasks increased following the intervention. - She spontaneously started playing games on her tablet for cognitive stimulation and using contacts, notebooks, photo, and recipe apps, without assistance. - She was proud of her tablet use and it had a positive impact on her confidence. - Caregiver burden remained absent or light throughout the study. |
| Köhler et al. (2021) Germany | Semi-structured interviews.  14 adults with mild cognitive impairment or mild to moderate dementia (AD), mean age 71 years, range 58 to 86 years.  (Amber). | To examine the mobility needs of people with mild to moderate dementia, and how these mobility needs can be supported by navigational assistance technology. | Any smartphone and tablet use. | Semi-structured interviews to explore living conditions and socioeconomic status, use of digital technology, need for help when navigating outside the home, and requirements for the usability of a device to support outdoor navigation. | - Nine participants would prefer their navigational assistive technology be on a smartphone, and one participant preferred a tablet computer. - Participants listed a reminder function, emergency calls, calendar, timetable for public transport and location display, watch, navigation, GPS, notes, and alarm function as important system functions. |
| Kwan et al. (2020) China | Cross-sectional trial with observations and qualitative interviews.  16 adults with dementia, median age 79 years, control group of 30 older adults without cognitive impairment, median age 67 years.  (Amber). | To explore the acceptability, feasibility and usability of older people with mild dementia to use smartphone for wayfinding. | iPhone. Map application (Apple Maps), voice command application (Siri). | Demographic data and data on cognitive function.  Performance on standardized wayfinding trial to assess efficacy of smartphone intervention.  Five markers to assess feasibility of smartphone intervention.  Questionnaire to assess acceptability of smartphone intervention.  Observations and post-intervention interviews to explore participants’ experience. | - All participants were able to successfully initiate Siri by voice on their first attempt. - Most participants were able to complete the wayfinding trial following the Maps application with no significant differences between groups. - Participants with mild dementia needed significantly more time to complete the training and wayfinding trials. - It was feasible and acceptable for people with dementia to use smartphones with voice navigation controls. |
| Routhier et al. (2012) Canada | Study 2: Single-case experimental study using AB design.  One adult with semantic dementia, age 51 years.  (Red). | To measure the effectiveness of a smartphone intervention to compensate for word comprehension and naming difficulties. | Smartphone. Internet application (Google Image), encyclopaedia application (Wikipedia). | Daily self-report data on smartphone use and ratings in the efficiency of the search and the ease of obtaining the desired information over study period (75 days). | - Participant was able to use search engines on Internet apps or encyclopaedia apps to find semantic information or pictures of words. - He frequently used the Internet app on his smartphone to prepare activities for work and used Internet dictionaries more than the paper dictionary. - He considered the smartphone fast, efficient, portable, accessible, and non-stigmatising compared to the paper dictionary. - His use of the smartphone over the weekend suggests that he enjoyed it and perceived its usefulness. |
| Scullin (2020) United States ^a^ | RCT.  52 adults with dementia or mild clinical impairment.  (N/A). | To investigate whether smartphone technology or a memory strategy can be used to assist participants with prospective memory tasks, reduce memory burden, and improve independent functioning in participants with mild AD. | Smartphone. Personal assistant application (Cortana). | Data on smartphone use and acceptance, cognition, memory, ADL and quality of life. | N/A |
| Wu et al. (2019) France | Cross-sectional survey.  323 adults with dementia (n=84), MCI (n=127) and older adults without cognitive impairment (n=112), mean age 76 years, categorised into 4 groups according to frequency of digital device use (daily use versus non-daily use).  (Amber). | To investigate cognitive function in relation to the use of a computer and a touchscreen device among older adults attending a memory clinic. | Any smartphone and tablet use. | Survey data on digital device use to categorise participants into groups according to frequency of digital device use.  Demographic data.  Data on cognitive function, short-term memory, working memory, processing speed, executive abilities, and episodic memory. | - Most participants used at least one type of digital device daily. Over a third used a touchscreen device daily and most of were also daily computer users. - Participants who used both a touchscreen device and a computer daily performed better on executive function and mental flexibility than other groups. - Participants with dementia who did not regularly use any digital device performed worse in several cognitive measures compared to participants using a digital device every day. |
| **No cognitive impairment (n=10)** | | | | | |
| Chan et al. (2016) United States | RCT.  54 older adults without cognitive impairment (mean age 75 years), split between iPad intervention group (n=18, mean age 75), social activities group (n=18, mean age 75) and placebo group (n=18, mean age 75).  (Amber). | To test whether older adults who were computer novices can be trained to become proficient users of a tablet computer using the iPad, which can be flexibly employed to perform many tasks associated with daily living. | iPad. iPad training intervention consisting of 2 2.5-hour training classes each week for 10 weeks and 10 hours of homework. | Data on processing speed, mental control, episodic memory and visuospatial processing at baseline and post-intervention. | - Improved performance on processing speed and memory in iPad intervention group compared with a social control and a placebo control. Although some individuals in the control groups also experienced some cognitive improvements, the iPad group showed significantly more improvement over time. |
| Gitlow (2014) United States | Non-experimental survey design.  82 older adults without cognitive impairment, mean age 79 years, range 60 to 97 years.  (Amber). | To investigate what types of technology older adults are using, what they are doing with these technologies, what they would like to be doing with technology and what barriers are preventing them from doing what they would like to do. | Any tablet use. | Data on customer needs categorised into sections concerning different technology types. | - 56% of older adults surveyed had cell phones. 17% had tablets. 35% of older adults did not need of want a cell phone; 49% for tablets. - The top three reasons for cell phones use were: personal calling (41%), voicemail (27%), and emergencies (26%). The top three reasons for tablet use were: emailing (7%), web browsing (5%), and contact information (4%). - For cell phones, 14% of participants wanted to learn to use appointment reminders and alarms, 9% wanted to use texting, and 7% wanted to use the calendar features. For tablets, 8% wanted to learn to use alarms and appointment reminders, 7% wanted to use shopping, and 4% wanted to use email and calendar features. |
| Nguyen et al. (2017) Australia | Cross-sectional survey.  153 older adults without cognitive impairment over 65 years.  (Amber). | To investigate how older people identify, select and learn to use mobile communication technologies to enhance communication and safety, and support independent living. | Any smartphone and tablet use. | Purpose-designed survey to gather information about opinions and experiences on mobile communication technologies as a means to support independent living. | - 84% of respondents were technology users but only 3.3% reported using a smartphone. - 44% of respondents were interested in trying out new products, devices, or services. - Devices were used for: emergencies or security purposes; to reachable by family and friends, and for a sense of safety. Fewer respondents would use devices for information services, work, recreation, or education. - 20% of respondents considered reminders as important to assist with daily life. - 18% of respondents considered assistance with navigation as important, with male respondents and those married/partnered more likely to use this function. |
| Petrovčič et al. (2019) Slovenia | Population-based survey.  1581 older adults without cognitive impairment, mean age 68 years, range 55 to 95 years. 640 older adults without cognitive impairment used in modelling, mean age 65 years, range 55 to 88 years.  (Amber). | To explore factors predicting seniors’ interest in using three different types of assistive apps. | Any application use. | Interest in seven assistive smartphone apps and questionnaire data on predictors of seniors’ interest and use of technology for independent living. | - Mobile phones were used by 90% of respondents, and 84% used them daily. 27% of mobile phone users used a smartphone. More than 81% respondents had heard about smartphones, with 61% among them having at least some familiarity with a smartphone. - Respondents were reluctant to adopt Internet-based mobile services and apps. One in four individuals over 55 years of age had never downloaded a smartphone application. - Respondents reported a high interest in an SOS button, followed by ICE contacts, fall detection, and GPS navigation. There was less interest in video calling, physical activity monitors, and medication reminders. |
| Rosales and Fernández-Ardèvol (2016) Spain ^c^ | Quantitative study tracking mobile app usage.  238 smartphone users, mean age 39 years, range 20 to 76 years.  Qualitative focus group study.  24 older adults without cognitive impairment, mean age 71 years, range 55 to 81 years.  (Amber). | To analyse the use of smartphones by older adults. | Any smartphone use. | Log data to indicate general patterns of smartphone usage across age ranges.  Focus group data to understand usage patterns from the perspective of older adult users’ reported experiences. | - Smartphones were a central part of participants’ everyday lives, even those who were initially reluctant to have a smartphone or were critical of others’ excessive smartphone use. - Personal information management apps were used more frequently by older participants. - Participants reported extensive use of note apps and calendar apps as memory aids. Some participants reported use of reminder apps. |
| Vaportzis et al. (2018) Scotland | Mixed methods study using post-intervention questionnaire.  43 older adults without cognitive impairment, mean age 69 years, range 55 to 76 years.  Post-intervention semi-structured focus group study.  14 older adults without cognitive impairment, mean age 68 years, range 65 to 75 years.  (Amber). | To investigate older adults’ experience in the “Tablet for Healthy Ageing” intervention program to understand what they found helpful or unhelpful about the tablet training intervention. | Tablet. | Questionnaire data to give opinions about tablets and applications.  Focus groups data to investigate older adults’ perception and attitudes toward tablet training following their participation in a tablet intervention. | - Participants were confident that tablet training could have beneficial effects on mental abilities. Some participants reported feeling cognitively faster and having better memory or reasoning skills. - Processing speed was significantly improved. - Half the group thought that a tablet could have positive effects on other aspects of health and wellbeing, such as active and healthy aging. - Most participants reported that it was likely or very likely that they would use a tablet in the future. |
| Vaportzis et al. (2017a) Scotland | Semi-structured focus group study.  18 older adults without cognitive impairment, mean age 71 years, range 65 to 75 years.  (Amber). | To investigate perceptions of, and barriers to, interacting with tablets in healthy older adults who were novice tablet users, and to explore the acceptability and usability of tablets as a potential tool to improve the health and wellbeing of older adults. | Tablets (Asus TF103CX, Asus Google Nexus, Samsung Galaxy Tab 3 8”, Samsung Galaxy Tab 3 10.1”, Apple iPad Mini). Maps application (Google Maps), news application (BBC News), Internet application (Google Chrome). | Focus group data to explore older adults’ perceptions and attitudes toward tablets and technology in general, and to refine a proposed intervention protocol for the “Tablets for Healthy Ageing” intervention program.  Tablet Experience Questionnaire data to rate experience with tablets, and to give opinions about tablets and applications. | - Participants wanted to use tablets to communicate better with younger generations. - Some participants believed that learning to use a tablet could improve various skills and abilities, such as faster cognition and keeping their brains active. - Others reported concerns that it would be harder to focus or that tablet use would deter them from using their memory because they would not need to remember events or facts. - Most participants enjoyed the tablet experience and said they were likely to use a tablet in the future. Half requested to be included in the “Tablet for Healthy Ageing” intervention program. |
| Vaportzis et al. (2017b) Scotland | Prospective RCT.  43 older adults without cognitive impairment, range 65 to 75 years, split between intervention group (n=22, mean age 68 years) and no-contact control group (n=21, mean age 70 years).  (Amber). | To test the efficacy of a tablet computer training intervention to improve cognitive abilities of older adults, and to investigate whether engaging with a new mentally challenging activity has cognitive benefits. | Tablet. Tablet training intervention consisting of a 2-hour class once a week for 10 consecutive weeks in addition to homework and encouragement to use the tablets as much as possible. | Data on cognitive function, verbal comprehension, perceptual processing, working memory, and processing speed.  Data related to psychological wellbeing, sleep patterns, social support, physical activities and other activities taken but not discussed. | - Improved performance on processing speed in intervention group. - No significant main effects or interactions for verbal comprehension, perceptual reasoning, or working memory. - For participants in the stroke group, the smartphone’s use as a memory and organisational aid was the most significant benefit. |
| Yuan et al. (2019) China | Cross-sectional survey.  2600 older adults without cognitive impairment, mean age 69 years, stratified by gender and categorised into 3 groups according to the number of smartphone functions used.  (Amber). | To investigate gender different in the use of smartphones and in cognitive ability, as well as the associations between smartphone use and general cognitive health and multi-domain cognitive health. | Any smartphone use. | Survey data on smartphone use to categorise participants according to number of smartphone functions used.  Demographic data to stratify data according to demographics.  Data on general and multi-domain cognitive health, including delayed memory, visuospatial ability, executive ability, attention, language, and orientation. | - Nearly 30% of the total participants were smartphone users, the majority of which were men. - Both male and female frequent smartphone users were more likely to attain higher scores in all cognitive subdomains than infrequent and non-smartphone users. - Use of more smartphone functions was positively associated with general cognitive health and all subdomains except memory and orientation. |
| Zilberman et al. (2016) United States | Pre-intervention and post-intervention semi-structured interviews.  8 older adults without cognitive impairment, mean age 68.  (Amber). | To evaluate changes in self-reported participation and satisfaction of performance of up to 5 (i)ADLs for older adults after an 8-week educational tablet-training program. | iPad Mini tablet, keyboard, case and stand. Tablet training intervention consisting of 1-hour educational session and 1-hour open laboratory session every week for 8 consecutive weeks. | Self-identified performance of (i)ADLs and occupational performance data at baseline and post-intervention to detect changes in self-perception of performance and satisfaction of self-identified problem areas.  Interview data from week 4 (mid-intervention) and week 8 (post-intervention) to investigate participants’ perceived successes of the intervention program. | - The ADL most frequently addressed by the tablet training intervention was functional mobility. Next was communication management, and then health management and maintenance, which included developing and managing routines for health and wellbeing promotion such as physical fitness and medication routines. - Other (i)ADLs: community mobility, financial management, shopping, and meal preparation and clean-up. - Participants’ perceived performance on the - (i)ADLs significantly improved following the intervention. Despite some initial difficulties, all the participants enjoyed using the tablet, used their tablet outside of the program, and would continue to use it in their daily lives. |

^a^ Protocol

^b^ Study was included because, although some participants were under 50 years old, the mean age of the sample was over 50 years old.

^c^ Study was included because, although the mean age of the quantitative study sample was under 50 years old, all qualitative study participants were over 50 years old.

Supplementary Table 3: Full summary of facilitators and barriers to smartphone and tablet use

| **Study; country** | **Barriers** | **Facilitators** |
| --- | --- | --- |
| **Acquired brain injury (n=8)** | | |
| Benge et al. (2020) United States | - Lower level of education - Older age - Presence or suspicion of geriatric cognitive disorder | - Higher level of education - Younger age |
| Bos et al. (2017) New Zealand ^b^ | - Difficult and frustrating to learn to use - Dislike of smartphone and reluctance to use smartphone features and applications | - Audible notifications - Convenience of multiple functions in one device - Engagement with smartphone training intervention - Interest and willingness to learn to use smartphone - Perception of smartphone and apps as useful and effective - Portability of device |
| Gustavsson et al. (2018) Sweden and Denmark ^b^ | - Post-stroke cognitive impairments - Post-stroke fine motor impairments | - Device use met need to manage everyday life - Feeling of connectedness with others - Feeling of safety - Enhanced independence and participation - Perception of smart devices as ordinary objects and non-stigmatising - Pre-existing familiarity with ICT |
| Lemke et al. (2019) New Zealand | - Difficult to learn to use - ICT functionality did not meet individual needs - Motor impairments - Perception of ICT use as “cheating” when compensating for stroke-induced impairments and memory - Physical features of device, e.g., size of devices, unfamiliar software (device-specific) - Sensory impairments - Speech impairments | - Convenience of small handheld devices in situations requiring mobility - Device use met individual needs - Feeling of connectedness with others - Feeling of safety - Motivation from family members and friends - Perception of device as non-stigmatising - Perception of device as useful and effective - Pre-existing familiarity with devices |
| Ramirez-Hernandez et al. (2021) Australia ^b^ | - Pre-existing strategies to aid memory, e.g., paper-based diaries, reminders from others | - Convenience of multiple functions in one device - Device use met individual needs - Engagement in smartphone training intervention - Enhanced self-efficacy - Motivation from family members and friends - Perception of smartphone as useful and effective - Portability of device - Pre-existing familiarity with smartphones |
| Ramirez-Hernandez et al. (2020) Australia ^a^ | N/A | N/A |
| Rivest et al. (2018) Canada | None reported. | - Engagement in smartphone training intervention - Enjoyment of learning something new - Gaining competence and sense of mastery of smartphone use |
| Wong et al. (2017) Australia | - Difficult to learn to use and remember how to use - Lack of instruction from rehabilitation clinicians - Motor impairments - Older age | - Ability to search for apps to meet individual needs - Access to the Internet - Convenience of small size of smartphone - Easy to use - Feeling of connectedness to others - Physical features of device (e.g., large easy to see display, long-lasting battery, touchscreen, small and lightweight hardware) - Portability of device |
| **Dementia or mild cognitive impairment (n=10)** | | |
| Bier et al. (2015) Canada | None reported. | - Pre-existing familiarity with Internet |
| Bier et al. (2018) Canada | None reported. | - Pre-existing familiarity with computers |
| El Haj et al. (2017) France | None reported. | - Perception of smartphone as discreet and non-stigmatising - Pre-existing familiarity with smartphone |
| El Haj et al. (2021) France | None reported. | - Interest and willingness to learn to use smartphone - Pre-existing familiarity with smartphone |
| Imbeault et al. (2018) Canada | - Difficult to learn to use - Overwhelming choice of devices | - Enjoyment of learning something new - Enhanced self-efficacy - Perception of tablet as useful |
| Köhler et al. (2021) Germany | - Fine motor impairments - Lack of knowledge and familiarity - Sensory impairments | - Convenience of multiple functions in one device - Perception of smartphone/tablet as ordinary objects and non-stigmatising |
| Kwan et al. (2020) China | - Cognitive impairments - iPhone interface busy and difficult to use (device-specific) - Sensory impairments - Technology anxiety - Weak or unreliable GPS signal (device-specific) | - Easy to use - Enhanced self-efficacy - Perception of smartphone as useful - Positive attitude towards technology use |
| Routhier et al. (2012) Canada | None reported. | - Easy to use - Perception of smartphone as fast, efficient, and useful - Perception of smartphone as non-stigmatising - Portability of device - Pre-existing familiarity with Internet and computers |
| Scullin and Jones (2020) United States ^a^ | N/A | N/A |
| Wu et al. (2019) France | - Lower level of education - Older age | - Higher level of education - Younger age |
| **No cognitive impairment (n=10)** | | |
| Chan et al. (2016) United States | None reported. | None reported. |
| Gitlow (2014) United States | - Cognitive impairments - Fine motor impairments - Lack of knowledge and familiarity | None reported. |
| Nguyen et al. (2017) Australia | - Cost of device - Difficult to learn to use - Lack of interest - Lack of knowledge and familiarity - Motor impairments - No perceived need - Overwhelming choice of devices and features - Physical features of device, e.g., small keys, small display text, low contrast (device-specific) - Lack of instruction manuals or user guides - Sensory impairments | - Easy to use - Younger age |
| Petrovčič et al. (2019) Slovenia | - Cognitive impairments - Dislike of smartphones from previous negative experiences - Higher socio-economic status - Living alone - Motor impairments - Sensory impairments - Technology anxiety | - Compatibility with lifestyle - Being in employment - Low smartphone anxiety - Older age - Perception of smartphone as useful - Presence of chronic health conditions |
| Rosales and Fernández-Ardèvol (2016) Spain ^c^ | - Confidence in existing cognitive and memory abilities - Pre-existing strategies to aid memory, e.g., paper-based diaries, reminders from others | - Enjoyment of learning something new |
| Vaportzis et al. (2018) Scotland | - Fear of technological addiction - Lack of confidence in tablet use - Very small physical features of tablet (device-specific) | - Easy to use - Feeling of connectedness with others - Perception of tablet as useful and enjoyable - Portability of device |
| Vaportzis et al. (2017a) Scotland | - Cost of tablets and additional equipment - Feelings of inadequacy in comparison to younger generations - Lack of confidence - Lack of instructions and guidance, or instructions too technical - Lack of knowledge - Motor impairments - Physical features of tablets, e.g., weight, cumbersome buttons (device-specific) - Sensory impairments | - Access to information - Interest in and willingness to adopt tablet - Portability of device - Versatility of tablet and apps |
| Vaportzis et al. (2017b) Scotland | See Vaportzis et al. (2018) | - See Vaportzis et al. (2018) |
| Yuan et al. (2019) China | - Sensory impairments - Technophobia | - Motivation from family members and friends |
| Zilberman et al. (2016) United States | - Difficult to learn to use | - Enjoyment of app use - Perceived usefulness of apps |

Supplementary Figure 1: Summary infographic of findings

**PRISMA 2020 Checklist**

| **Section and Topic** | **Item #** | **Checklist item** | **Location where item is reported** |
| --- | --- | --- | --- |
| **TITLE** | | |  |
| Title | 1 | Identify the report as a systematic review. | Page 2 |
| **ABSTRACT** | | |  |
| Abstract | 2 | See the PRISMA 2020 for Abstracts checklist. | Page 2 |
| **INTRODUCTION** | | |  |
| Rationale | 3 | Describe the rationale for the review in the context of existing knowledge. | Pages 4-6 |
| Objectives | 4 | Provide an explicit statement of the objective(s) or question(s) the review addresses. | Page 6 |
| **METHODS** | | |  |
| Eligibility criteria | 5 | Specify the inclusion and exclusion criteria for the review and how studies were grouped for the syntheses. | Pages 6-8 |
| Information sources | 6 | Specify all databases, registers, websites, organisations, reference lists and other sources searched or consulted to identify studies. Specify the date when each source was last searched or consulted. | Page 8 |
| Search strategy | 7 | Present the full search strategies for all databases, registers and websites, including any filters and limits used. | Pages 8, 49 |
| Selection process | 8 | Specify the methods used to decide whether a study met the inclusion criteria of the review, including how many reviewers screened each record and each report retrieved, whether they worked independently, and if applicable, details of automation tools used in the process. | Pages 8-9 |
| Data collection process | 9 | Specify the methods used to collect data from reports, including how many reviewers collected data from each report, whether they worked independently, any processes for obtaining or confirming data from study investigators, and if applicable, details of automation tools used in the process. | Page 9 |
| Data items | 10a | List and define all outcomes for which data were sought. Specify whether all results that were compatible with each outcome domain in each study were sought (e.g. for all measures, time points, analyses), and if not, the methods used to decide which results to collect. | Page 9 |
|  | 10b | List and define all other variables for which data were sought (e.g. participant and intervention characteristics, funding sources). Describe any assumptions made about any missing or unclear information. | Page 9 |
| Study risk of bias assessment | 11 | Specify the methods used to assess risk of bias in the included studies, including details of the tool(s) used, how many reviewers assessed each study and whether they worked independently, and if applicable, details of automation tools used in the process. | Pages 8-10 |
| Effect measures | 12 | Specify for each outcome the effect measure(s) (e.g. risk ratio, mean difference) used in the synthesis or presentation of results. | N/A |
| Synthesis methods | 13a | Describe the processes used to decide which studies were eligible for each synthesis (e.g. tabulating the study intervention characteristics and comparing against the planned groups for each synthesis (item #5)). | Pages 6-10 |
|  | 13b | Describe any methods required to prepare the data for presentation or synthesis, such as handling of missing summary statistics, or data conversions. | N/A |
|  | 13c | Describe any methods used to tabulate or visually display results of individual studies and syntheses. | N/A |
|  | 13d | Describe any methods used to synthesize results and provide a rationale for the choice(s). If meta-analysis was performed, describe the model(s), method(s) to identify the presence and extent of statistical heterogeneity, and software package(s) used. | Pages 8-10 |
|  | 13e | Describe any methods used to explore possible causes of heterogeneity among study results (e.g. subgroup analysis, meta-regression). | N/A |
|  | 13f | Describe any sensitivity analyses conducted to assess robustness of the synthesized results. | N/A |
| Reporting bias assessment | 14 | Describe any methods used to assess risk of bias due to missing results in a synthesis (arising from reporting biases). | N/A |
| Certainty assessment | 15 | Describe any methods used to assess certainty (or confidence) in the body of evidence for an outcome. | Pages 8-9 |
| **RESULTS** | | |  |
| Study selection | 16a | Describe the results of the search and selection process, from the number of records identified in the search to the number of studies included in the review, ideally using a flow diagram. | Pages 9, 34 |
|  | 16b | Cite studies that might appear to meet the inclusion criteria, but which were excluded, and explain why they were excluded. | N/A |
| Study characteristics | 17 | Cite each included study and present its characteristics. | Pages 27-32, 35-44 |
| Risk of bias in studies | 18 | Present assessments of risk of bias for each included study. | Pages 9-10 |
| Results of individual studies | 19 | For all outcomes, present, for each study: (a) summary statistics for each group (where appropriate) and (b) an effect estimate and its precision (e.g. confidence/credible interval), ideally using structured tables or plots. | N/A |
| Results of syntheses | 20a | For each synthesis, briefly summarise the characteristics and risk of bias among contributing studies. | Pages 10-16 |
|  | 20b | Present results of all statistical syntheses conducted. If meta-analysis was done, present for each the summary estimate and its precision (e.g. confidence/credible interval) and measures of statistical heterogeneity. If comparing groups, describe the direction of the effect. | N/A |
|  | 20c | Present results of all investigations of possible causes of heterogeneity among study results. | N/A |
|  | 20d | Present results of all sensitivity analyses conducted to assess the robustness of the synthesized results. | N/A |
| Reporting biases | 21 | Present assessments of risk of bias due to missing results (arising from reporting biases) for each synthesis assessed. | N/A |
| Certainty of evidence | 22 | Present assessments of certainty (or confidence) in the body of evidence for each outcome assessed. | N/A |
| **DISCUSSION** | | |  |
| Discussion | 23a | Provide a general interpretation of the results in the context of other evidence. | Pages 16-18 |
|  | 23b | Discuss any limitations of the evidence included in the review. | Pages 18-20 |
|  | 23c | Discuss any limitations of the review processes used. | Pages 18-20 |
|  | 23d | Discuss implications of the results for practice, policy, and future research. | Pages 16-20 |
| **OTHER INFORMATION** | | |  |
| Registration and protocol | 24a | Provide registration information for the review, including register name and registration number, or state that the review was not registered. | Page 6 |
|  | 24b | Indicate where the review protocol can be accessed, or state that a protocol was not prepared. | Page 6 |
|  | 24c | Describe and explain any amendments to information provided at registration or in the protocol. | N/A |
| Support | 25 | Describe sources of financial or non-financial support for the review, and the role of the funders or sponsors in the review. | Page 3 |
| Competing interests | 26 | Declare any competing interests of review authors. | Page 3 |
| Availability of data, code and other materials | 27 | Report which of the following are publicly available and where they can be found: template data collection forms; data extracted from included studies; data used for all analyses; analytic code; any other materials used in the review. | Pages 8, 9,35-49 |

*From:*  Page, M.J., McKenzie, J.E., Bossuyt, P.M., Boutron, I., Hoffmann, T.C., Mulrow, C.D., Shamseer, L., Tetzlaff, J.M., Akl, E.A., Brennan, S.E., Chou, R., Glanville, J., Grimshaw, J.M., Hrobjartsson, A., Lalu, M.M., Li, T., Loder, E.W., Mayo-Wilson, E., McDonald, S., McGuinness, L.A., Stewart, L.A., Thomas, J., Tricco, A.C., Welch, V.A., Whiting, P., & Moher, D. (2020). The PRISMA 2020 statement: An updated guideline for reporting systematic reviews. *BMJ* 2021, 372, n71. doi: 10.1136/bmj.n71
